# IKK16 and SGI1027 induce cytoplasmic vacuolization, and their combination with chemotherapy drugs can enhance the efficacy of cancer therapeutics

**DOI:** 10.1038/s41419-026-09142-w

**Published:** 2026-08-05

**Authors:** Arwa Alsubait, Bandar Alghanem, Hemza Ghadbane, Sarah Huwaizi, Khawlah Almuhalhil, Feras Almourfi, Abdulelah Altolayyan, Abdullah Alanazi, Bader Tayeb Hazazi, Mohamed Boudjelal, Rizwan Ali

**Affiliations:** 1 Medical Research Core Facility and Platforms (MRCFP), King Abdullah International Medical Research Center (KAIMRC), King Saud bin Abdulaziz University for Health Sciences, Ministry of National Guard Health Affairs (MNGHA), Riyadh, Saudi Arabia; 2 Department of Pathology and Laboratory Medicine, King Abdullah Medical City (KAMC), MNGHA, Riyadh, Saudi Arabia

**Keywords:** High-throughput screening, Apoptosis, Breast cancer

## Abstract

Cell death represents a critical process in the maintenance of cellular homeostasis. Paraptosis, a mechanism of programmed cell death, is distinguished by extensive cytoplasmic vacuolization. We have identified two commercially available compounds, IKK16 and SGI1027, which can induce cytoplasmic vacuolization leading to paraptosis in cancer cells. We have characterized and studied various cancer cell lines by applying molecular biology, proteomics, gene expression analyses, and microscopy-based techniques. Additionally, our gene expression analyses indicate that GADD45A, and NPC2 genes may serve as potential markers for identifying paraptosis, and validated their involvement in IKK16/SGI1027-mediated paraptosis with a cellular functional assay. Furthermore, the proteomics data revealed a novel set of proteins previously unassociated with paraptosis. We have substantiated our proteomics findings through western blotting and immunocytochemistry in breast and colorectal cancer cell lines, demonstrating similar effects. Moreover, we have shown that combining paraptosis-inducing compounds with anti-cancer drugs can reduce the required overall dosage concentration while preserving therapeutic efficacy, potentially revolutionizing cancer treatment.

**Figure:**
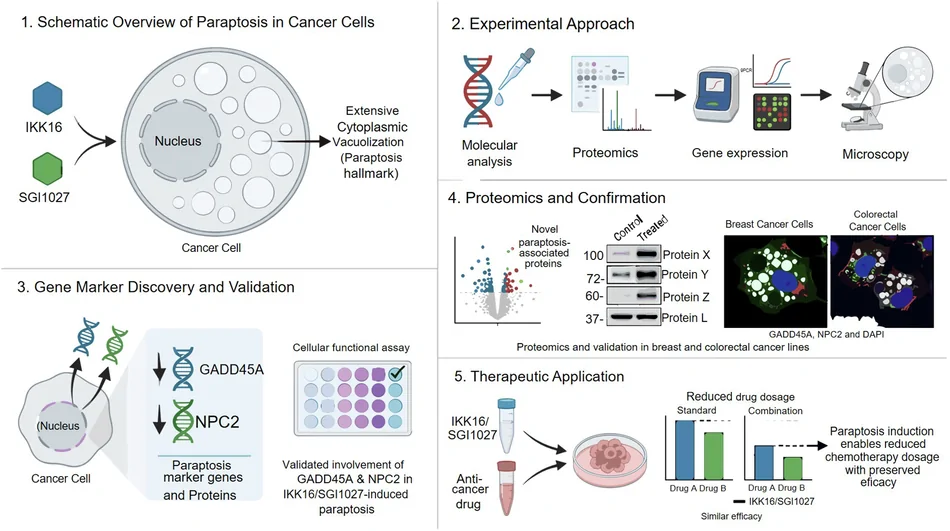

## Introduction

Cell death is broadly categorized into programmed and non-programmed mechanisms. Non-programmed cell death, such as necrosis, can be triggered independently or as a consequence of programmed cell death failure. In contrast, programmed cell death (PCD) involves a highly controlled cascade of molecular events [1]. PCD is a vital cellular function required to maintain physiological homeostasis, control cellular populations, and eliminate unwanted or infected cells [2]. Consequently, aberrant PCD mechanisms frequently result in uncontrolled cell growth and malignant transformation. Morphologically, PCD is characterized by membrane blebbing, cell shrinkage, nuclear condensation, and genomic fragmentation. Investigating the distinctive molecular mechanisms of different PCD pathways has revealed essential targets for novel drug development [3].

To date, known PCD mechanisms include apoptosis, necroptosis, paraptosis, pyroptosis, ferroptosis, and autophagy [3]. Among these, apoptosis is the most extensively characterized pathway and has served as a primary target in oncology drug discovery for the past two decades [4]. These diverse cell death mechanisms play critical roles in modulating the immunosuppressive tumor microenvironment and determining the efficacy of anti-cancer therapies. Interestingly, PCD can exert dual roles; depending on the specific intracellular context and the nature of the released damage-associated molecular patterns, it can either promote tumorigenesis or induce anti-cancer activity [5].

The targeted regulation of PCD using small molecules has demonstrated significant therapeutic efficacy in cancer treatment [6]. For example, ampelopsin promotes both apoptosis and autophagy-dependent cell death pathways in glioma cells via the generation of reactive oxygen species (ROS) and the activation of c-Jun N-terminal kinase (JNK) [7]. Similarly, shikonin is a promising anti-cancer compound that activates JNK signaling to induce apoptosis and autophagy in colorectal cancer cells [8]. While conventional anti-cancer therapies suppress tumorigenesis primarily via apoptosis, tumor cells frequently develop resistance by evolving mechanisms to escape apoptotic stimuli. Therefore, identifying alternative, non-apoptotic PCD pathways is critical for developing next-generation targeted cancer therapeutics.Paraptosis is a relatively novel and understudied form of PCD. It is morphologically characterized by endoplasmic reticulum (ER) dilation, mitochondrial swelling, and extensive cytoplasmic vacuolization, without the typical features of apoptosis or necrosis. The term “paraptosis” was first coined in 2000 by Sperandio et al., who observed this phenotype in mouse fibroblasts and human cancer cell lines overexpressing the insulin-like growth factor 1 receptor (IGF1R) [9]. Recent studies indicate that various small molecules can initiate paraptosis in diverse tumor cell lines. However, the precise role of paraptosis in oncology remains fully elucidated due to a limited understanding of its upstream molecular cascades and a lack of paraptosis-specific biochemical markers [10]. Notably, apoptosis-resistant cancer cells often retain sensitivity to alternative PCD pathways, suggesting that paraptosis can be successfully exploited to eliminate chemoresistant malignancies [11].

Here, we report for the first time that IKK16, a selective inhibitor of IκB kinase (IKK) [12], and SGI1027, a DNA methyltransferase (DNMT) inhibitor [13], induce potent cytoplasmic vacuolization in cancer cells at submicromolar concentrations, ultimately culminating in paraptotic cell death. IKK16 inhibits IKK in the nanomolar range, blocking TNF-α-stimulated IκB degradation and the subsequent expression of adhesion molecules, E-selectin, ICAM, and VCAM [14]. Invivo, it inhibits LPS-induced TNF-α release and neutrophil extravasation in thioglycollate-induced peritonitis [15]. Whereas, SGI1027 has been used as a DNMT inhibitor to study its effects on gene expression during osteogenic differentiation [16]. It has also been used as a DNMT 3a inhibitor to study its effects on interleukin-6 (IL-6)-induced loss of forkhead box P3 (Foxp3) gene expression in B-lymphocyte-induced maturation protein 1 (Blimp1)-Treg cells [17]. SGI1027 directly inhibits DNMT activity by competing with the cofactor S-adenosylmethionine (SAM) in the methylation reaction [18]. SGI1027 treatment of cancer cell lines induced degradation of DNMT1, but not DNMT3A or DNMT3B, and in RKO cells, caused re-expression of the silenced tumor suppressor genes p16, MLH1, and TIMP3 [19].

To evaluate the therapeutic potential of paraptosis induction by these compounds, we utilized breast and colorectal cancer cell lines due to their high global prevalence [20, 21]. Breast cancer remains a profound global burden, ranking first in worldwide cancer incidence, and maintains high recurrence and mortality rates despite conventional treatment interventions [22]. Similarly, colorectal carcinoma is the second most lethal cancer globally, with projections suggesting its global incidence will double by 2035 [23]. Overcoming therapeutic resistance is one of the greatest challenges in modern oncology. Combination regimens enhance efficacy by maximizing cancer cell destruction, minimizing the emergence of resistance, and reducing off-target side effects.

In this study, we combined these novel paraptosis-inducing agents, IKK16 and SGI1027, with the conventional anthracycline and anthracenedione chemotherapeutic agents mitoxantrone, doxorubicin, and daunorubicin to combat apoptotic resistance. Mechanistically, our findings reveal that these agents trigger paraptotic cell death concurrent with a significant downregulation of the stress-responsive gene GADD45A and the endolysosomal cholesterol transporter protein NPC2. This molecular axis highlights an unexpected intersection between biochemical homeostatic stress and intracellular lipid trafficking during non-apoptotic cell death. Identifying these new paraptosis-inducing dynamics expands our fundamental understanding of alternative cell death pathways and facilitates the discovery of novel therapeutic combination strategies for cancer prevention and treatment.

## Materials and methods

### Cell lines culture

Breast cancer cell line KAIMRC2 [24] was isolated and characterized in King Abdullah International Medical Research Center (KAIMRC), Riyadh, Saudi Arabia. The breast cancer cell line MDAMB231 and colorectal cancer cell line HCT8 were purchased from ATCC, USA. Patient-derived primary breast fibroblast cells were isolated from patients after consenting and instutional review board (IRB) approval. All the cells were maintained in advanced Dulbecco’s Modified Eagle Medium (DMEM) (Invitrogen) supplemented with 10% fetal bovine serum (FBS), 50 units/ml penicillin and 50 µg/ml streptomycin (Gibco), and 2 mM L-glutamine (Gibco) at 37 ° C in a humidified 5% CO_2_ atmosphere.

### 3D spheroids cell culture

KAIMRC1 spheroids [25] are 3D cell cultures derived from the naturally immortalized human breast carcinoma cell line, KAIMRC1 [26]. Spheroid formation is rapidly induced by supplementing Advanced DMEM with 10% Newborn Calf Serum (NBCS) in 96-well transparent white opaque plates. The following day, the cell culture plates were monitored under a microscope to verify spheroid formation before any downstream experiments. Post-combination treatment, the CellTiter-Glo 3D Cell Viability Assay (Promega) was used to measure cell survival in 3D spheroids.

### Compound treatment

To determine the dose-response effect, Mitoxantrone dihydrochloride (Cat. # 4250), IKK16 (Cat. # 2539), and SGI1027 (Cat. # 5155) were used. To rule out other well-known cell death pathways, such as Apoptosis, Autophagy, and Necroptosis, Actinomycin D (Cat.# 1229), Z Vad FMK (Cat.# 2163), Bafilomycin A1 (Cat.# 1334), and Necrostatin-1 (Cat.# 2324), Taxol (Cat.# 1097), and Staurosporine (Cat.# 1285) were used, respectively. All compounds were purchased from Tocris, USA. Compounds were serially diluted two-folds in DMSO per well starting from 100 µM concentration in an opaque-walled 96-well plate (Costar). This plate served as a master plate. 10,000 cells/well in 100 μl were seeded in clear transparent walled 96-well plates in complete media. Cells were incubated at 37 °C humidified incubator overnight before treatment.

### Cell morphology

To evaluate the morphological changes in cancer cells, transmitted light and phase-contrast images were captured using a Zeiss LSM 780 microscope system. The images were processed in the Zen Black edition.

### Immunocytochemistry (ICC)

Cells were seeded 24 h before the experiment in black 96-well plates, as described above in the cell culture section. They were then fixed, permeabilized, and incubated overnight at 4 °C with the appropriate antibodies (Table 1). To detect the nucleus, counterstaining was performed with HOECHST 33342 (2.5 µg/ml, Life Technologies, USA). All the stainings were performed in triplicates. The plate was imaged using the Zeiss cLSM 780 Microscope System (Fig. 7a). The data was analyzed using the Zen Black edition and Microsoft Excel software.

**Table 1:** Antibodies for ICC.

	Antibodies	Manufacturer	Catalog #	IF Conc.	Host
1	ALIX	Thermo Fischer Scientific	MA183977	1:100 Primary	Mouse
2	Olig2		MA515810		Mouse
3	CD133 (5E3)		MA5-18323		Mouse
4	GLUT1 (SA0377)		MA5-31960		Rabbit
5	LSS		PA5-56912		Rabbit
6	FDFT1 (OTI1H9)		MA5-25675		Mouse
7	Annexin α6		720161		Rabbit
8	SCD (CD.E10)		MA5-27542		Mouse
9	ALDH1B1		PA5-54314		Rabbit
10	GDF15 (CL0328)		MA5-31346		Mouse
11	IDI1		PA5-21720		Rabbit
12	SQSTM1 (GT1478)		MA5-27800		Mouse
13	Annexin α2 (Z014)		03-4400		Mouse
14	PTMA		PA5-71580		Rabbit
15	LC3A/LC3B		PA116931		Rabbit
16	PARP		MA5-15607		Mouse
17	Alexa fluor 488, anti-mouse IgG(H + L)		A28175	1:400 Secondary	Goat
18	Alexa fluor 488, anti-rabbit IgG (H + L)		A11034		Goat

### Light microscopy

Cells were seeded 24 h before the experiment in μ- slide 8-well Ibitreat chamber slides (Cat. # 80826, Ibidi, Germany). In the case of time-lapse imaging, cells were treated with the drugs and imaged using cLSM 780 (Carl Zeiss, Germany) using transmitted light. In some cases, the EVOS FL Auto microscope (Molecular Devices) was also used to acquire transmitted light images.

For fluorescence imaging, cells were treated for 24 h with IKK16 (Cat.# 2539), SGI1027 (Cat.# 5155), Taxol (Paclitaxel, Cat.# 1097), Mitoxantrone, Staurosporine (Cat.# 1285), Daunorubicin hydrochloride (Cat.# 1467), Doxorubicin (Cat.# 2252), and Etoposide (Cat.# 1226), at 3.12 µM concentration, all purchased from Tocris. Post-treatment, cells were stained, and Calcein AM (2 µg/mL) was detected using Argon laser at 488 nm/520–530 (ex/em), HOECHST33342 (2.5 µg/mL) was detected using UV laser at 350 nm/460 nm (ex/em), and Propidium Iodide (PI) (2.5 µg/mL) was detected using HeNe laser at 633 nm/650 (ex/em).

### High content imaging (HCI) assay for ROS activity

KAIMRC2 cells were plated in 96-well plates at a density of 10,000 cells per well, treated with 3.12 µM of IKK16 and 6.2 µM of SGI1027, and incubated for 24 h. Post-treatment, cells were stained with Dihydroethidium (DHE) purchased from ThermoFisher Scientific, Cat# D11347 at a concentration of 1:1000 for 30 min. Plates were imaged using Molecular Devices ImageXpress® Micro and analyzed using MetaXpress® software, Molecular Devices, USA. The multiwavelength cell scoring analysis module available in the MetaXpress software was used to count cells positive for DHE. The acquired values were fed into the Microsoft Excel software to generate a graph of the percentage of ROS-positive cells. All experiments were performed in triplicates.

### Fluorescence spectroscopy for mitochondrial membrane potential (MMP) and ROS assays

Cells were grown as described above and treated with desired concentrations of IKK16, SGI1027, Mitoxantrone, and Daunorubicin, with incubation for 24 h. Post-treatment, cells were stained with JC-1 (Cat# T3168) for the MMP assay. Dihydroethidium (DHE) was purchased from ThermoFisher Scientific (Cat# D11347) and used in the oxidative stress ROS assay at a concentration of 1:1000 for 20 min. Plates were read using Molecular Devices Spectramax M5 and later imaged with Molecular Devices ImageXpress Micro for high content imaging. The acquired values were fed into the Microsoft Excel software to generate the graphs. All experiments were performed in triplicates.

### Western blot

Lysates from the KAIMRC2, MDAMB231 (not shown), and HCT8 (not shown) cell lines were prepared, and protein levels were determined using the BCA method (Thermofisher Scientific). Protein supernatants were boiled in a loading buffer, separated by 10% SDS-PAGE, and transferred to polyvinylidene difluoride (PVDF) transfer membranes. The blots were blocked for 1 h with 5% BSA in PBS-T at room temperature and then incubated with specific primary antibodies (Table 2) overnight at 4 °C. After washing with PBS-T, the membranes were incubated with HRP-conjugated secondary antibodies at room temperature for 1 h. Finally, an enhanced chemiluminescent (ECL) substrate was used for band detection using a ChemiDoc MP System (Bio-Rad, USA) and analyzed on ImageLab and MS Excel 2013 software (Fig. 4d).

**Table 2:** Antibodies for WB.

	Antibodies	Manufacturer	Catalog #	WB Conc.	Host
1	CD133 (5E3)	Thermo Fischer Scientific	MA5-18323	1:1000 Primary	Mouse
2	LSS		PA5-56912		Rabbit
3	FDFT1 (OTI1H9)		MA5-25675		Mouse
4	ALDH1B1		PA5-54314		Rabbit
5	GDF15 (CL0328)		MA5-31346		Mouse
6	SQSTM1 (GT1478)		MA5-27800		Mouse
7	Annexin α2 (Z014)		03-4400		Mouse
8	CASP3	Molecules-ON	AB-M-052		Mouse
9	CASP8		AB-M-053		Mouse
10	JUN		AB-M-055		Mouse
11	PhosphoPTEN (S380)		AB-M-056		Mouse
12	ESRRA		AB-M-058		Mouse
13	CCND1		AB-M-057		Mouse
14	ERBB2		AB-M-059		Mouse
15	CARD9		AB-M-060		Mouse
16	Cav-1		AB-M-051		Mouse
17	Anti-Rabbit IgG, HRP	Cell Signaling	7074S	1:2000 Secondary	Goat
18	Anti-Mouse IgG, HRP		7076S		Goat

### Sample preparation and analysis of real-time qPCR

Total RNA was isolated from all the cells using the RNA Isolation Kit (Ambion) following manufacturers’ instructions. RNA was quantified on a Nanodrop 8000 Spectrophotometer (Thermofischer Scientific, USA). Applied Biosystems’ high-capacity cDNA reverse transcription kit was used to reverse transcribe cDNA. Commercially available Human Apoptosis (PAHS-012Z), Cell Death (PAHS-212Z), Stress and toxicity (PAHS-003Z), and Osmotic stress (PAHS-151Z) RT [2] Profiler PCR Arrays (Applied Biosystems^®^, USA) were employed. The samples were processed following the manufacturer’s protocol. PCR amplification was performed on a 7900HT Fast Real-time PCR System (Applied Biosystems^®^, USA). Data were analyzed on RQ Manager 1.2.1 software (Applied Biosystems^®^, USA). Microsoft Excel 2013 was used for further analysis, and the ΔΔ_Ct_ method was utilized to calculate relative changes in mRNA expression levels (Fig. 5).

### MTT cell proliferation assay

KAIMRC2 cells were seeded at 10,000 cells/well into 96-well plates and incubated at 5% CO_2_ incubator overnight. Subsequently, cells were treated with compounds in serial dilution ranging from 0 to 100 µM for 48 h. Later, the MTT assay followed the standard protocol, and the absorbance was read at 560 nm using a Molecular Devices Spectra Max Plus 384 Spectrophotometer. Background readings were collected at 620 nm. Absorbance readings were normalized and expressed as a relative percentage. The data were analyzed in Microsoft Excel and GraphPad Prism software, and the half-maximal inhibitory concentration (IC_50_) was determined (Figs. 2c, d, and 8a, b). Error bars denote standard deviation (SD). The statistical significance of differences was determined using the Student’s t-test. *p* < 0.05 was considered statistically significant.

### Human protein profiler arrays

KAIMRC2 cells were subjected to Human Proteome Profiler™ (R&D Systems) antibody arrays to identify the proteins involved in apoptosis and paraptosis. The Human Apoptosis Array (Cat.# ARY009) and Human Cell Stress Array (Cat.# ARY018) were purchased from R&D Systems, Minneapolis, USA, and used according to the manufacturer’s protocol. KAIMRC2 cells were seeded overnight in 6-well plates in complete advanced DMEM. Later, cells were treated with IKK16, SGI1027, Mitoxantrone, and vehicle for 24 h. Cells were lysed, and the lysate was processed for both profilers. The membranes were imaged using ImageLab software, a ChemiDoc MP System (Bio-Rad), and the data were quantified by Gel Analyzer and ImageJ software (NIH, USA) (Fig. 4 c–e).

### Transmission electron microscopy (TEM)

KAIMRC2, MDAMB231, HCT8, and primary fibroblast cells were treated with either IKK16, SGI1027, Mitoxantrone, or DMSO as a control. Following treatment, the cells were fixed with 4% glutaraldehyde (Cat.# G5882, Sigma-Aldrich, Darmstadt, Germany) in 0.1 M PBS (pH 7.4) for 1 h at 4 °C. The samples were then fixed with 1% Osmium tetroxide (OsO_4_) in 0.1 M PBS (pH 7.4) at room temperature for 1 h, followed by dehydration in a graded concentration of acetone and infiltration with acetone: resin at 1:1 for 1 h, and then at 1:2 for 3 h. The embedding was in epoxy resin (Araldite) and polymerized overnight at 80 °C. Later, ultrathin sections of the specimen were obtained with an ultramicrotome, mounted on copper grids, and stained for contrast with uranyl acetate and lead citrate. Images were acquired on a JEOL-JEM 1400 (JEOL Ltd, USA) operating at 120 kV using a Gatan camera with digital micrograph imaging software.

### Scanning electron microscopy (SEM)

Cells were grown and fixed as described above. Fixed cells were dehydrated with graded concentrations of Ethanol (Sigma) and transferred to appropriate carbon taped stubs (Ted Pella, USA) for SEM. To enhance electron conductivity, samples were coated with gold/palladium (Au/Pd) by sputter coating and examined using an FEI NanoSEM 450 scanning electron microscope operating at 10 kV.

### Proteomics sample preparation

One hundred micrograms of protein from each sample was isolated through precipitation with methanol/chloroform/water. The resulting protein pellets were then redissolved in a buffer containing Urea and Tris-HCl, followed by reduction with 500 mM Tris (2-carboxyethyl) phosphine hydrochloride and alkylation with 500 mM 2-chloroacetamide. Protein samples were digested overnight with trypsin into peptides. The digestion was halted using formic acid.

### NanoLC-MS/MS analysis

Three replicates of each sample were analyzed using Ekspert NanoLC 400 (Eksigent, Dublin, CA, USA) coupled to a TripleTOF 5600 (Sciex, Concord, Canada) in Data-Dependent Acquisition (DDA) mode. Peptides were first trapped on a C18 trap column before separation on a C18 Nano-LC column (3C18-CL, 75 µm × 15 cm, Eksigent, USA) using a gradient elution of 5-80% acetonitrile with 0.1% formic acid. The eluted peptides were then analyzed by MS1 (400–2000 *m/z*) and MS2 (400-1250 *m/z*) with the top 10 most intense precursors ions (+2 to +5) exceeding 200 cps selected for fragmentation using rolling collision energy with a collision energy spread of 5 eV. Instrumental parameters were set as GS1 = 25, GS2 = 0, CUR = 25, ISVF = 2500, and IHT = 60.

### Proteomics data analysis

Proteins were identified using ProteinPilot Software (v4.5, Sciex). The MS/MS data was searched against a human protein database downloaded on October 29, 2019, with a 1.3 unused protscore confidence threshold. False discovery rate (FDR) was analyzed to ensure confidence in protein identification. Protein quantification was then performed with Skyline software (v 23.1.0.268). VolcaNoseR was used for the volcano plot visualization [27]. Pathway analysis was performed by ShinyGO 0.80 [28].

### Generation of cells overexpressing GADD45A and NPC2

Cells were transfected using FuGENE® HD Transfection Reagent (Promega, WI, USA) according to the manufacturer’s instructions. In brief, freshly passaged adhered cells at approximately 70–80% confluency in 96-well plates were transfected with 200–400 ng plasmid DNA and 0.5 µL FuGENE HD reagent per well. Overexpression plasmids for GADD45A (EF1A::GADD45A + GFP-P2A-PuroR), and NPC2 (EF1A::NPC2 + mCherry-P2A-HygroR) were synthesized and sequence-validated (VectorBuilder Inc., IL, USA). At 48–72 h post-transfection, antibiotic selection was initiated: cells transfected with GADD45A overexression plasmid were selected with 1 µg/mL Puromycin, whereas cells transfected with NPC2 overexpression plasmid were selected with 1 µg/mL Hygromycin B to generate stable lines. The cells were maintained under continuous selection and expanded, while green and red fluorescence were monitored to confirm successful plasmid integration.

### Statistical analyses

The Student’s t-test or one-way analysis of variance (ANOVA) was used to conduct differential comparisons between two or multiple groups, respectively. Tukey’s test was used to compare groups using one-way ANOVA. GraphPad Prism (version 8.0) was used for statistical analyses. Statistical significance was set at a *P* value of <0.05. *p*-values ≥ 0.1 = NS, 0.05–0.02 = *, 0.01–0.002 = ** and ≤0.001 = ***.

## Results

### Characterization of paraptosis induction

To evaluate the therapeutic potential of exploiting alternative programmed cell death pathways, we treated breast and colorectal cancer cell lines with the IκB kinase inhibitor IKK16 and the DNMT inhibitor SGI1027to explore the onset of cytoplasmic vacuolization leading to paraptosis.

### IKK16 and SGI1027 induce cytoplasmic vacuolization in various cell lines

**Fig. 1:**
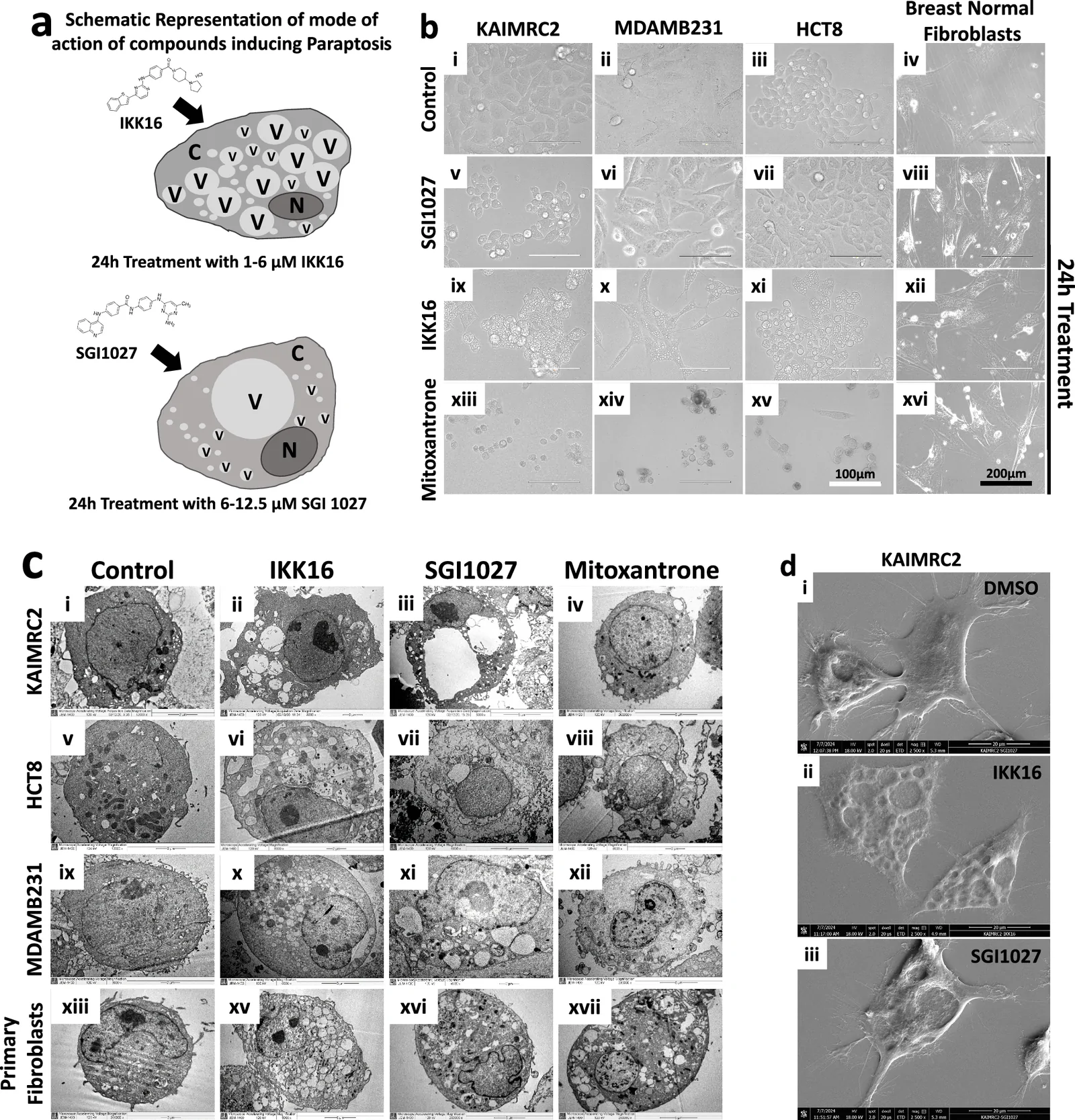
Characterization of cytoplasmic vacuolization induced by IKK16 and SGI1027 in cancer and normal fibroblast cells. **a** A schematic representation of cell treatment with IKK16 and SGI1027 highlights the cells’ fate. N: indicates nucleus. V: indicates cytoplasmic vacuoles. C: indicates cytoplasm. **b** Cancer (KAIMRC2, MDAMB231, HCT8) and breast normal fibroblast cells were treated with 6.25 µM of IKK16, 12.5 µM SGI1027, and 6.25 µM Mitoxantrone for 24 h, and the control cells were treated with DMSO. Phase contrast microscopic images were acquired to visualize changes in cell morphology. The white scale bar represents 100 µm, and the black scale bar represents 200 µm. **c** TEM observations of KAIMRC2, HCT8, MDAMBB231, and Primary normal fibroblasts treated with IKK16 or SGI1027. Treated cells exhibited cytoplasmic vacuoles, a characteristic of paraptosis not seen in control cells. Mitoxantrone was used as an apoptosis inducer. Scale bar= 2–5 µm. **d** SEM Images of (i) DMSO, (ii) IKK16, and (iii) SGI1027 treated KAIMRC2 cell line.

**Fig. 2:**
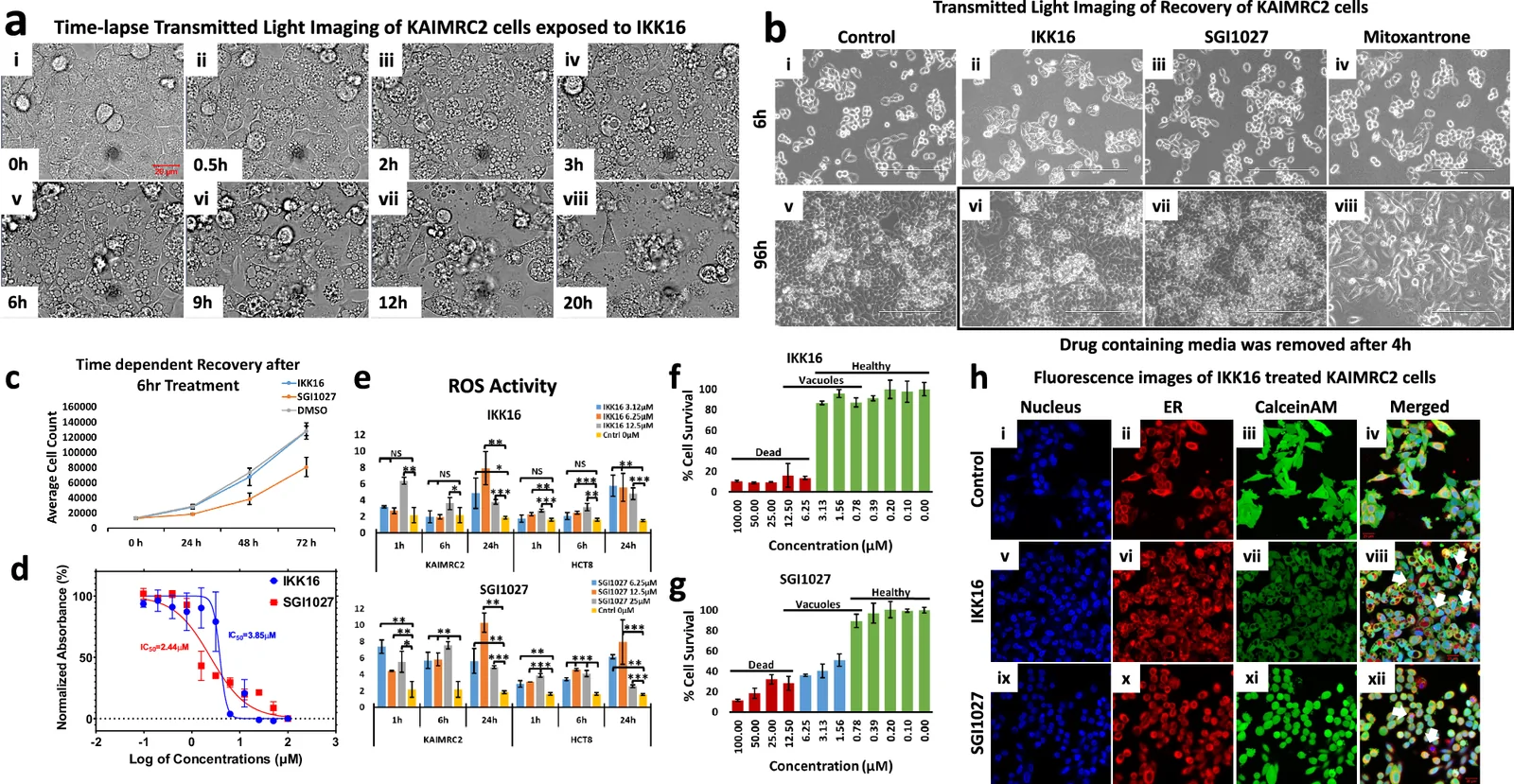
IKK16 and SGI1027 induce cytoplasmic vacuolization and paraptosis-like death in KAIMRC2. **a** Time-lapse Imaging of paraptosis induction in KAIMRC2 cells after treatment with IKK16 at 12.5 µM concentration. Transmitted light images were acquired at 0 h, 0.5 h, 2 h, 3 h, 6 h, 9 h, 12 h, and 20 h). It was noted that the higher concentration of IKK16 induces paraptosis within 2 h, and the cells start to die at ~12 h. Whereas lower concentrations do not induce cell death. Scale bar = 20 µm. **b** Treatment and recovery of KAIMRC2 cells post 6 h of treatment with IKK16 and SGI1027. Scale bar = 200 µm. **c** Graphical representation of Time-dependent recovery monitored for 72 h. **d** Graphical representation of MTT assay results showing IC_50_ values for IKK16 and SGI1027. (**e**) ROS activity of KAIMRC2 and HCT8 cell lines. The cells were treated with IKK16 and SGI1027 for 1 h, 6 h and 24 h. The cells were then stained with DHE for 20 min. A significant increase in the production of ROS was noticed in both IKK16 and SGI1027 treated cells especially after 24 h. **f**, **g** Graphical representation of % of cell survival at different concentrations of compounds (0–100 µM). **h** Fluorescence micrographs of untreated\treated KAIMRC2 cells with IKK16 or SGI1027 for 24 h. The cells were stained for nucleus (HOECHST 33342, blue), endoplasmic reticulum (ER tracker, Red), and viable cells (CalceinAM, green). The white arrowheads show dilated ER stained with ER Tracker Red—scale bar = 100 µm. *p*-values ≥ 0.1 = NS, 0.05–0.02 = *, 0.01–0.002 = ** and ≤0.001 = ***.

Cell vacuolization often accompanies cell death, and it is a hallmark of paraptosis [29]. We observed the morphological changes in the cancer cell lines (KAIMRC2, MDAMB31, and HCT8) and the breast normal fibroblast cells under a phase-contrast microscope after the treatment with compounds IKK16, SGI1027, and Mitoxantrone (Fig. 1b). Both IKK16 and SGI1027 induced cytoplasmic vacuolization in all the cells. Whereas mitoxantrone induced apoptotic cell death. After confirming that different cell types show paraptosis in response to these two compounds, we focused only on KAIMRC2 for our downstream experiments. We noticed that, unlike the morphological characteristics of apoptosis or any other cell death mechanism, cancer and normal fibroblast cells formed numerous cytoplasmic vacuoles upon the treatment with IKK16 and SGI1027. The half-maximal inhibitory concentration (IC_50_) values were acquired for IKK16 and SGI1027 (Fig. 2d). Figure 2f, g showcases the range of concentrations that induce vacuolation in the cells without killing. The treatment concentration range was 3–12 µM for 24 h (Fig. 2a). Interestingly, we noticed that exposure to 3 µM IKK16 resulted in many small cytoplasmic vacuoles in the cells within 3 h (Fig. 2aii–iv). These vacuoles dilated and covered most of the cytoplasmic space within 24 h of exposure. Similar phenomena were observed in cells treated with SGI1027 (Data not shown). However, SGI1027 treatment formed enlarged vacuoles that accumulated towards the cells’ periphery (Fig. 1bv, diii), suggesting a slightly different mode of action than IKK16. Transmission and scanning electron microscopy (TEM & SEM) validated light microscopy results (Fig. 1c, d). Further, TEM images revealed the dilation of the endoplasmic reticulum (ER), mitochondria, and double membrane of the nucleus, especially in IKK16-treated cells (Fig. 4b*, orange arrows*). Fluorescence microscopy of treated KAIMRC2 cells further corroborated the dilation of ER (Fig. 2hviii, xii, *white* *arrows*). Here, the cells were stained with ER Tracker Red. The images show most vacuoles with red punctuate staining, which is not the case in the control cells (Fig. 2hiv).

### Mitochondrial membrane potential during cytoplasmic vacuolization

**Fig. 3:**
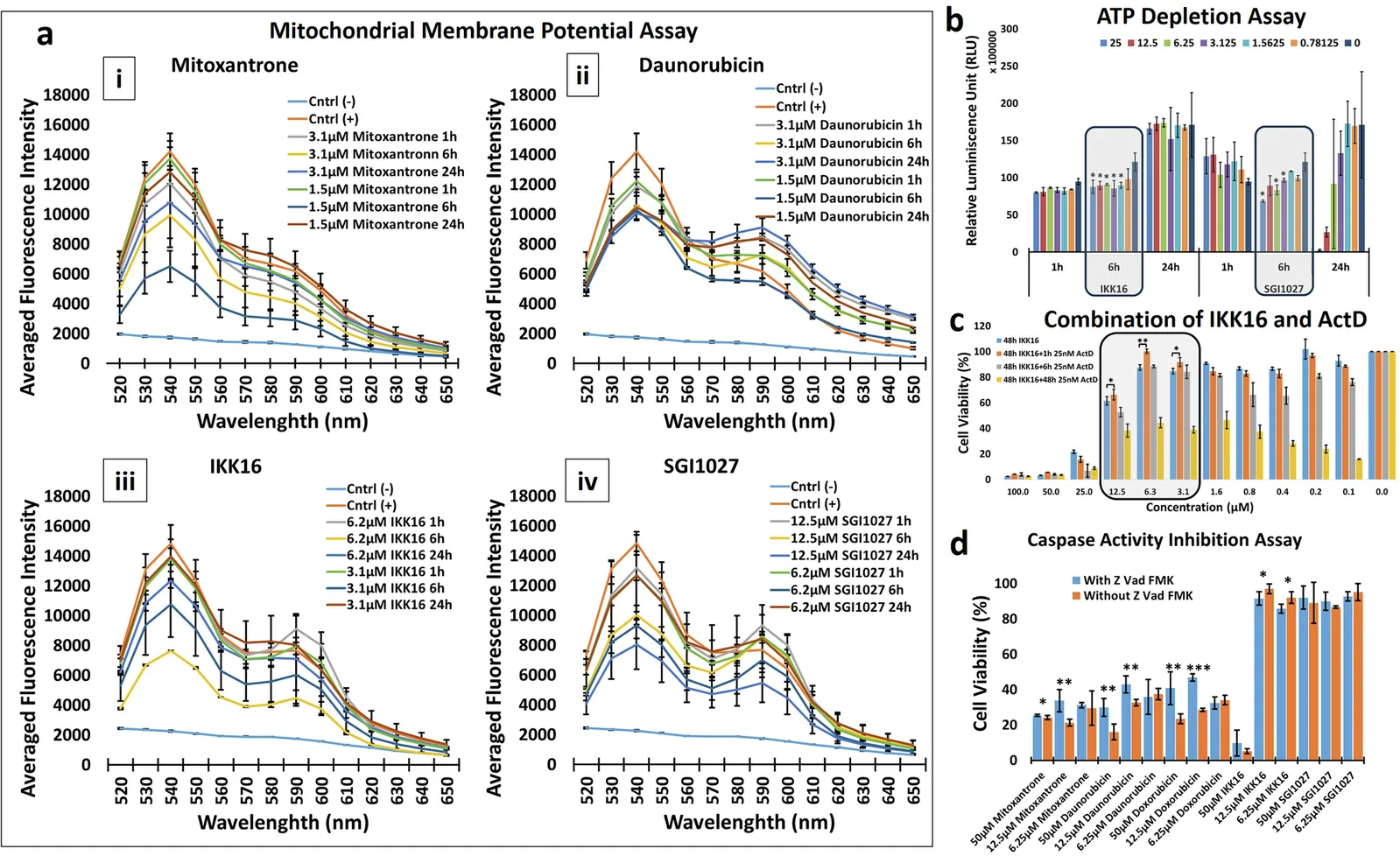
Biochemical and Bioenergetic Assays . **a** Mitochondrial membrane potential assay. The KAIMRC2 cell line was treated with IKK16, SGI1027, Daunorubicin, and Mitoxantrone at different concentrations for 1 h, 6 h and 24 h. Live cells were stained with JC1 and subjected to spectroscopy. IKK16, SGI1027, and Daunorubicin showed an increase in membrane potential indicated by the formation of mitochondrial aggregates at 590 nm (mostly at 1 h time point). **b** ATP depletion assay. The KAIMRC2 cell line was treated with IKK16 and SGI1027 for 1 h, 6 h, and 24 h. Maximum ATP depletion was observed at 6 h time point. **c** Cell viability of the KAIMRC2 cell line after combination treatment with IKK16. Increase in cell viability when cells were treated with 25 nM ActD for 1 h only at IKK16 concentrations of 12.5, 6.25, and 3.13 µM. **d** Caspase Activity Inhibition assay. A clear increase in cell viability in Mitoxantrone, Doxorubicin, and Daunorubicin can be seen upon treatment with 50 nM Z Vad FMK. In contrast, no increase in cell viability was observed when IKK16 and SGI1027 were used. It indicates that both drugs’ mode of action is Caspase-independent. *P*-values 0.05–0.02 = *, 0.01–0.002 = ** and ≤0.001 = ***.

JC-1 dye is used in apoptosis studies to monitor mitochondrial health. It accumulates in mitochondria based on potential; at low concentrations or membrane potential, it appears as monomers, emitting green fluorescence at 530 nm and accumulated J aggregates at 590 nm. JC-1 staining of cells treated with graded concentrations of IKK16, SGI1027, and Daunorubicin exhibited a time-dependent reduction in the red/green fluorescence intensity ratio, indicating depolarization of mitochondrial membrane and cytoplasmic diffusion of green monomers. In contrast, Mitoxantrone did not show any increase in J-aggregates (Fig. 3a). It is known that loss of mitochondrial membrane potential hinders ATP synthesis and results in an increase in ROS production [30].

### Reactive oxygen species (ROS) activity

ROS levels are known to be increased along with ER stress during paraptosis [29]. Cells were stained with DHE to determine the levels of ROS in KAIMRC2 and HCT8 cell lines treated with IKK16 and SGI1027 at increasing concentrations and time points (Fig. 2e). HCI was utilized to perform this experiment. The mean fluorescence intensity (MFI) was obtained by dividing the total fluorescence by the total number of cells to get the average fluorescence intensity per cell. The results showed a significant increase in ROS activity with increasing concentrations of compounds in both the cell lines. Interstingly, at 24 h timepoint the highest concetration of the compounds has decreased ROS activity than the lower concentrations. It is might be due to the toxicity of the higher concentrations of the compounds. Another intereting observation is the similar pattern of ROS induction in the cells after treatment with IKK16 and SGI1027. It indicates that both the compounds have similar mode of action when it comes to the induction of ROS.

### ATP depletion during cytoplasmic vacuolization

Next, we check the levels of ATP in the cells after treatment with IKK16 and SGI1027. Earlier, it was demonstrated that the depletion of ATP in paraptotic cells increases tumor immunity [31]. The cells were treated with graded concentrations of IKK16 and SGI1027 for three different time points. The titerglo assay was performed to assess the levels of ATP in and around the cells. In Fig. 3b, we show that there was a significant decrease in ATP levels in the cells, especially at the 6 h time point.

### Differentiation between paraptosis, apoptosis and other cell death mechanisms

**Fig. 4:**
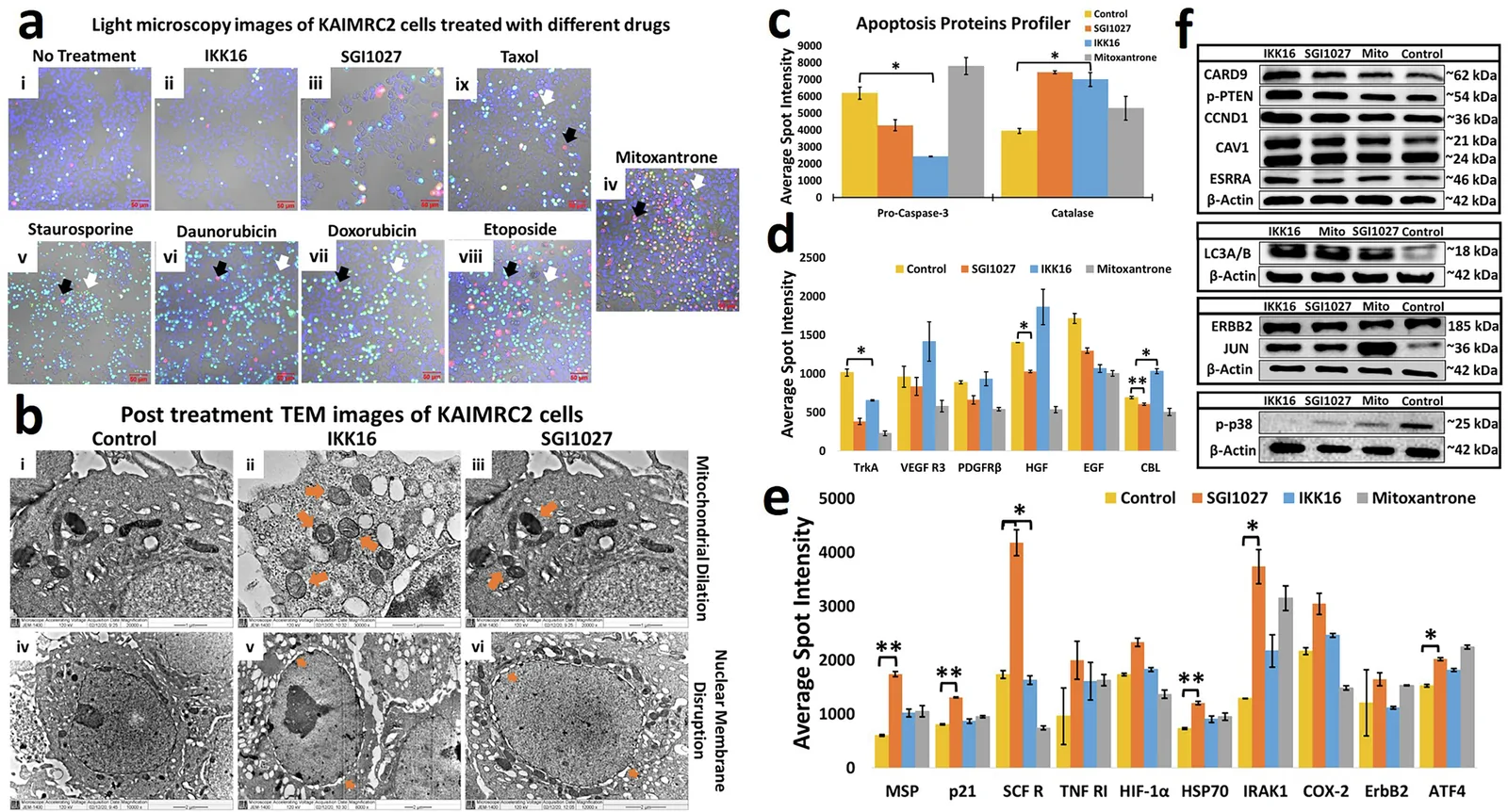
Microscopical imaging and protein profiling of KAIMRC2 paraptotic cells. **a** Fluorescence images of viable/dead staining of KAIMRC2 cells exposed to IKK16 and other drugs are known to induce apoptosis. The cells were stained with Hoechst 33342 (Blue) for the Nucleus, PI (Red) for dead cells, and YoPro-1 (Green) for apoptotic cells. The white arrowheads points to the apoptotic cells in the images wheras, the black arrowhead show dead cells. Scale bar 50 µm. **b** TEM images of KAIMRC2 cells treated with IKK16, SGI1027, or untreated for 24 h. Orange arrows highlighted morphological changes in cells that undergo paraptosis, including cytoplasmic vacuoles, mitochondrial dilation, and nuclear membrane disruption. Scale bar = 1–2 µm. **c**–**e** Apoptosis and Cell stress pathway protein profilers revealed several key proteins involved during paraptosis. **f** Representative western blots for LC3 A/B, p-p38, CARD9, p-PTEN, CCND1, CAV1, ESRRA, ERBB, and JUN using cell lysates of KAIMRC2 cells untreated or treated with IKK16, SGI1027, or Mitoxantrone. β-actin was used as a loading control. *P*-values 0.05–0.02 = *, 0.01–0.002 = ** and ≤0.001 = ***.

To differentiate between paraptotic and apoptotic cell death, we examined the expression of key proteins implicated in apoptotic cell death, such as caspase recruitment domain-containing protein 9 (CARD9), CASPASE 3 (CASP3), CASPASE 8 (CASP8), phosphorylated phosphatase, and tensin homolog (p-PTEN), Cyclin D1 (CCND1), Caveolin 1 (CAV1), Estrogen related receptor alpha (ESRRA), Receptor tyrosine kinase (ERBB2), and Jun proto-oncogene, AP-1 transcription factor subunit (JUN). The cells were treated with IKK16, SGI1027, apoptosis-inducing Mitoxantrone, and no treatment control. CARD9 overexpression in cells is known to decrease apoptosis [32]. P-38 is a known activator of apoptosis [33]. Upregulation of Cyclin D1 mediates apoptosis resistance through molecular chaperones [34]. Zou et al. [35], have shown that CAV1 acts as an anti-apoptotic protein. Whereas there are reports that show that CAV1, ERBB2, and PTEN promote apoptosis in certain conditions. Nevertheless, in our opinion, the combination of different regulatory pathways is responsible for the onset of a programmed cell death. That is why the combination of regulation of these reported proteins prevents apoptosis and promotes cell vacuolization, leading to paraptosis at higher concentrations of the compounds. An overexpression of CARD9, CCND1, CAV1, and JUN was observed after treatment with both compounds compared to untreated cells (Fig. 4f). Interestingly, p38 is highly downregulated hinting at the induction of the non-apoptotic pathway in the cells. c-JUN acts as a suppressor of PTEN [36]. It is possible that the increase in PTEN triggered the upregulation of JUN.

Next, we treated the cells with Z Vad FMK, a pan-caspase inhibitor [37]. Our results show that combining Mitoxantrone, Daunorubicin, and Doxorubicin with Z Vad FMK increased cell survival. In contrast, the combination of IKK16 and SGI1027 with Z Vad FMK did not show any difference in the cell proliferation rate of the cells (Fig. 3d). This indicated that both drugs use a caspase-independent pathway to cell death.

Further, we treated the cells with Actinomycin D (ActD), a known potent apoptosis inducer. It has also been used in a few cases to eliminate paraptosis [38]. Interestingly, when the cells were treated with 25 nM ActD for 1 h and then left incubated with IKK16 for another 48 h, an increase in cell viability was observed. 6 h incubation did not show any increase in cell viability. In contrast, 48 h incubation with both IKK16 and ActD showed a stark decrease in cell viability depicting the induction of apoptosis in the cells (Fig. 3c). Autophagy and paraptosis are two related but distinct cellular processes that help maintain cellular health and regulate cell death. Autophagy primarily serves as a survival strategy by recycling damaged cellular components, whereas paraptosis is a form of programmed cell death characterized by marked intracellular swelling.To rule out the possibility that IKK16 and SGI 1027 induce autophagy, we treated cells with the autophagy inhibitor Bafilomycin A1 (BafA1), a potent, reversible inhibitor of the vacuolar-type H^+^-ATPase. BafA1 partially rescued a few cells from vacuolation after IKK16 or SGI1027 treatment (Fig. 8n, o), which indicates the presence of acidic vacuoles alongside ER lumen dilation and marked mitochondrial swelling (Figs. 2h and 4b).

Further to rule out Necroptosis onset, we treated cells with Necrostatin-1 (Nec-1), a necrosis inhibitor. Nec1 rescues cells from the severe morphological destruction typical of necroptotic cell death, characterized by cellular swelling and membrane rupture. In our experiments, Nec1 was not able to inhibit vacuole formation in SGI1027 and/or IKK16-treated cells (Fig. 8k, l).

One of the main morphological characteristics of paraptosis is the appearance of cytoplasmic vacuoles. Therefore, we question whether IKK16 and SGI1027 triggered cell death via paraptosis. It is well known that the LC3 protein has an essential role in forming cytoplasmic vacuoles, and LC3 has been extensively used as a marker for paraptosis. Therefore, the levels of LC3 in KAIMRC2 were determined by western blotting and fluorescence microscopy (Figs. 4f and 7axxxi, xxxii). Western blotting and fluorescence microscopy results indicate increased LC3 protein expression after treatment with IKK16 and SGI1027. Next, we utilized apoptosis and cell stress protein profilers to determine the expression of essential proteins implicated in cell death pathways. The data revealed differential expression of several proteins. IRAK1, ATF4, HSP70, Catalase, MSP, p21, and SCFR were significantly upregulated, whereas Caspase-3 and TrkA were downregulated after exposure to both compounds (Fig. 4c–e). Most of these proteins are implicated in cell survival and proliferation. Caspase-3 is a frequently activated death protease, and Catalase overexpression decreases Caspase-3 activation [39].

Next, we examined the differences among other cell death mechanisms, such as autophagy and necroptosis.

**Fig. 5:**
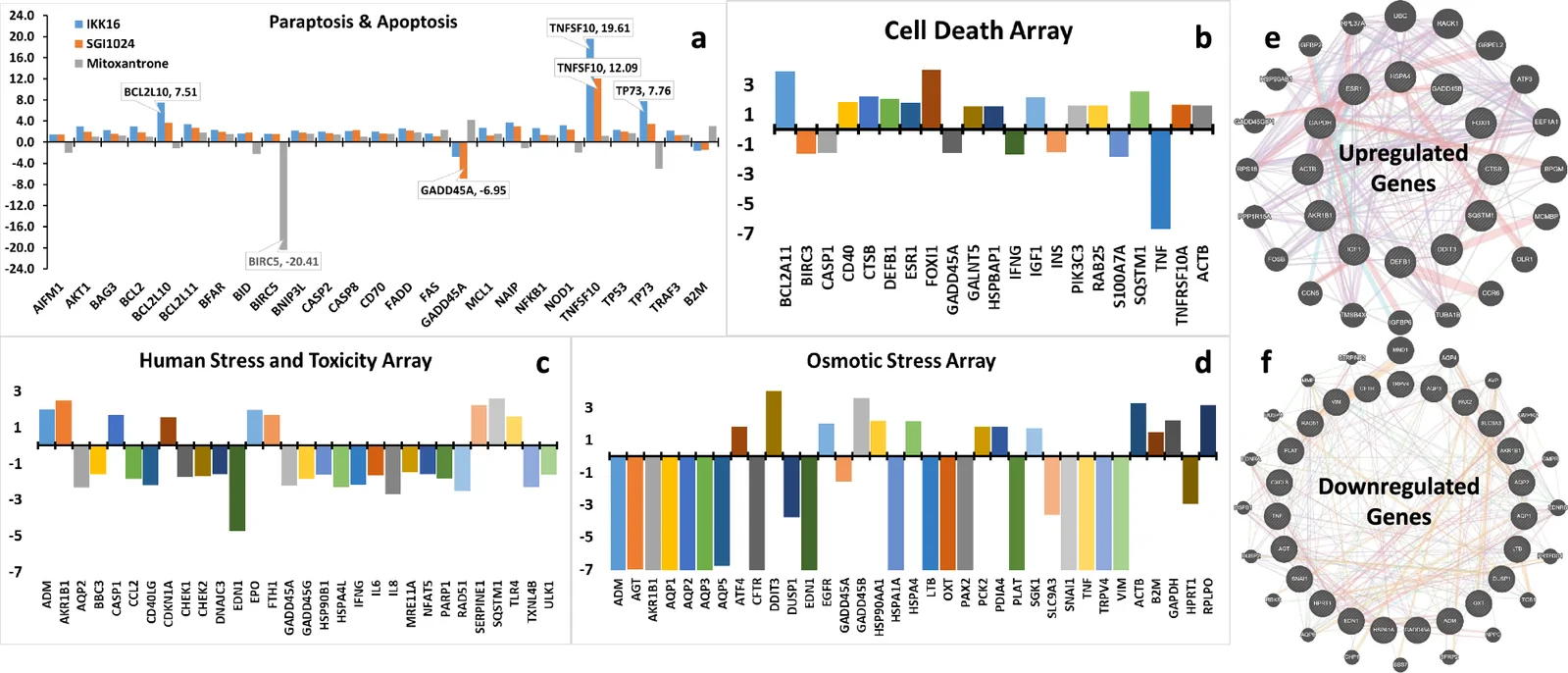
Gene expression profiling of KAIMRC2 paraptotic cells. **a** Different gene expression profiling was seen in cells treated with IKK16 and SGI1027 compared to apoptosis-inducing Mitoxantrone. **b**–**d** Human cell death, stress and toxicity, and osmotic stress arrays were utilized to uncover many genes noticeably affected during paraptosis. The results are depicted as fold change determined from the average difference change and made relative to untreated cells. **e** Markedly up-regulated genes network generated using Cytoscape. **f** Markedly down-regulated genes network generated using Cytoscape.

**Fig. 6:**
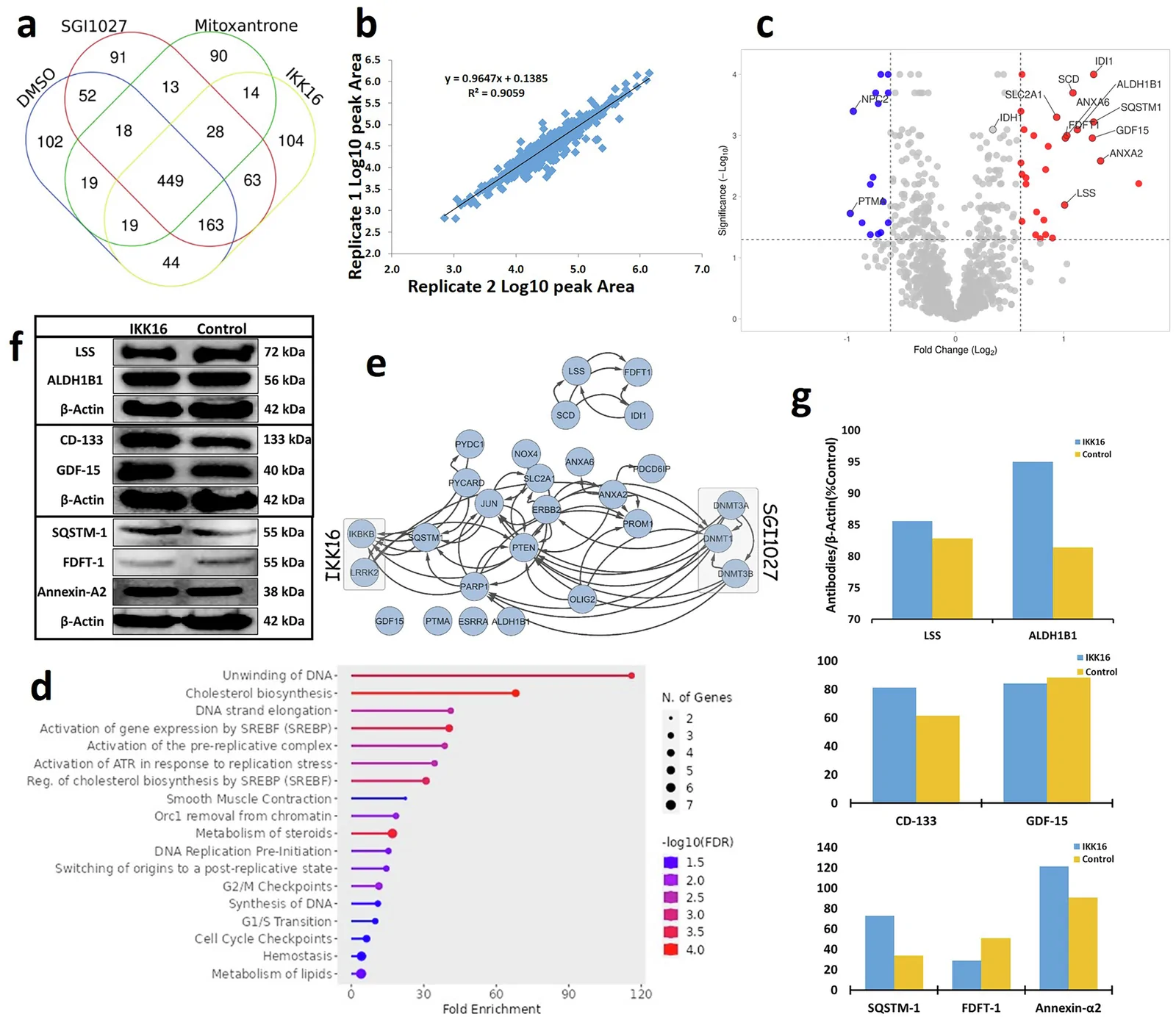
Quantitative proteomics analysis. **a** Venn diagram depicting identified proteins’ uniqueness and overlap across DMSO, IKK16, Mitoxantrone, and SGI1027 samples. **b** The scatter plot visualizes the reproducibility of protein intensity measurements across two replicate experiments performed on IKK16 samples. **c** Volcano plot of differentially expressed proteins in IKK16 versus DMSO. Red dots represent upregulated proteins (fold change ≥1.5), blue downregulated proteins (fold change ≤0.66), and gray dots are not significant and not regulated proteins. *p*-value significance level was set as 0.05. **d** Pathway enrichment analysis for IKK16/DMSO was performed using the ShinyGO tool with the Reactome database. **e** String network of identified proteins using Cytoscape. **f** Representative western blots of identified proteins for KAIMRC2 cells treated with IKK 16 or DMSO. The relative expression of the following proteins was determined: LSS, ALDH1β1, CD-133, GDF-15, SQSTM1, FDFT-1, and Annexin α2. β-actin was used as a loading control. **g** Graphical representation of the western blot bands.

**Fig. 7:**
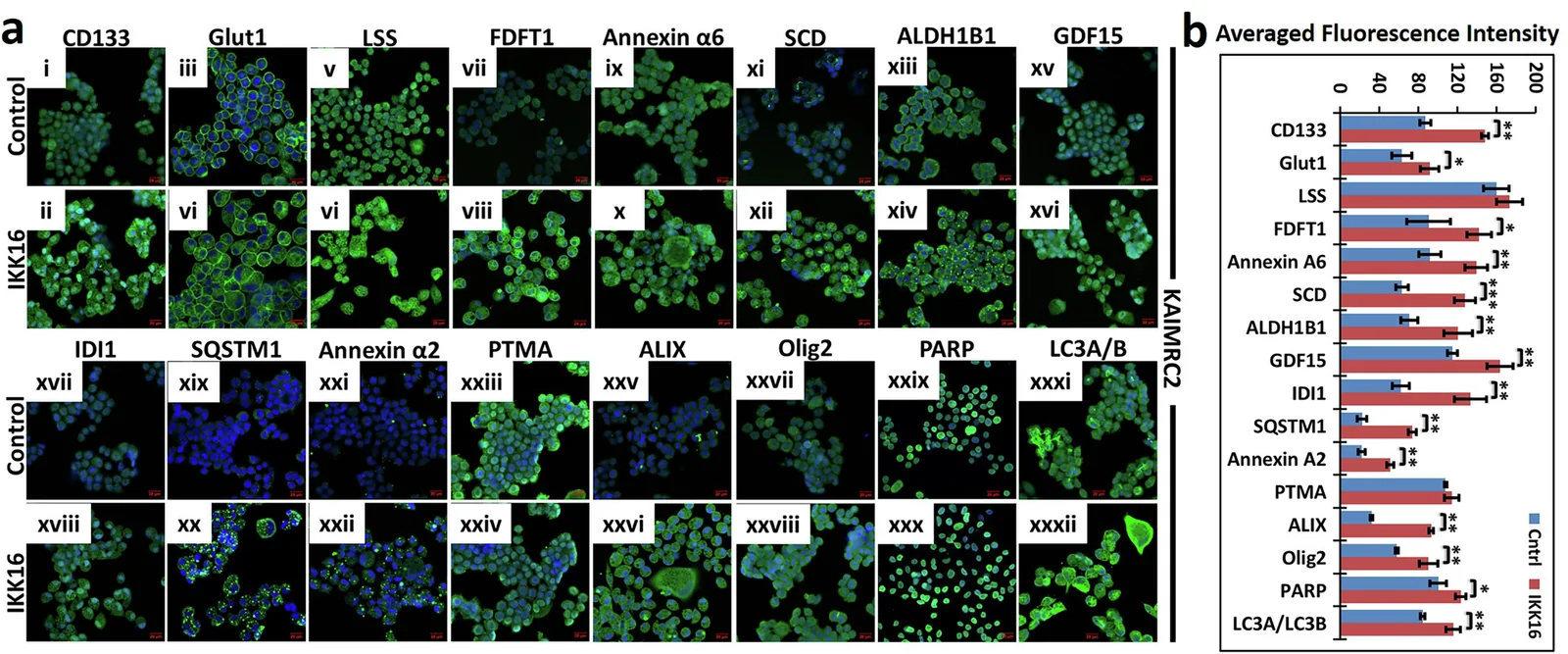
Validation of proteomic data by immunocytochemistry. **a** cLSM images of KAIMRC2 cells stained for selected protein candidates. The protein of interest is shown in green, and the nucleus is shown in blue. The images show staining of the following proteins: CD133, Glut1, LSS, FDFT1, Annexin α6, SCD, ALDH1B1, GDF15, IDI1, SQSTM1, Annexin α2, PTMA, ALIX, Olig2, PARP, LC3A/B (**b**) Graphical representation of relative expression for candidate proteins using averaged fluorescence intensities. *P*-values: *=0.05, **=0.01, ***=0.005.

The SQSTM1 (p62) gene, which encodes a receptor for selective autophagy, is upregulated when cells are treated with IKK16, as shown in our gene expression data (Fig. 5b, c). Moreover, its protein expression is also significantly upregulated (Figs. 6f and 7). The upregulation of this gene may serve as a negative regulator by activating pathways that inhibit autophagy [40].

Another gene, ULK1, a key regulator of autophagy, activates and phosphorylates other proteins during this process [41]. However, our Human Stress and Toxicity gene array data (Fig. 5c) show downregulation of ULK1 expression when cells are treated with IKK16.

NPC2 gene, a critical component in cellular homeostasis [42], encodes a soluble lysosomal protein that functions as a key cholesterol transporter. Naturally occurring mutations in NPC2 have been associated with Niemann-Pick disease type C2, a lysosomal storage disorder characterized by intracellular accumulation of unesterified cholesterol and other lipids. Beyond its lipid trafficking role, NPC2 dysfunction can disrupt lysosomal integrity, trigger endoplasmic reticulum stress, impair autophagic flux, and promote oxidative damage, thereby sensitizing cells to regulated cell death [43, 44]. In addition, altered NPC2 activity has been linked to apoptotic signaling and paraptosis-like vacuole formation, pointing to the possible contribution of cholesterol imbalance and lysosomal dysfunction to cell death mechanisms.

### Recovery of cancer cells from paraptosis

To assess the ability of IKK16 to induce cytoplasmic vacuolation, time-lapse imaging was conducted in KAIMRC2 cells. The cells were treated with IKK16 and monitored for 24 h (Fig. 2a). Cytoplasmic vacuoles were observed 30 min after exposure to the compound, and an increase in vacuoles and dead cells was observed over time. In addition, to determine if cancer cells can recover from paraptosis, KAIMRC2 cells were treated with 3.12 µM of IKK16 and 6.25 µM of SGI1027 for 4 h (Fig. 2b). Later, when the cytoplasmic vacuoles were visible, the drug-containing media was removed and replaced with fresh media. Interestingly, the cells started growing back after a phase of the initial shock. The cells began growing normally within 72 h, and 96 h post-exposure, the cytoplasmic vacuoles disappeared (Fig. 2c). Mitoxantrone was used as a positive control for apoptosis, showing cell growth arrest and that the cells did not grow backeven after the removal of the drug.

Transmission Electron Microscopy (TEM) evaluated the morphological changes in KAIMRC2 cells after exposure to IKK16 and SGI1027 for 24 h. Untreated KAIMRC2 cells showed normal morphology with intact membrane, scant cytoplasm, and round nuclei. However, exposure to IKK16 or SGI1027 induced the formation of cytoplasmic vacuoles, mitochondrial dilation, and nuclear membrane disruption (Fig. 4b, orange arrows), which are markers of paraptosis. To confirm our findings, fluorescence microscopy was used to observe KAIMRC2 cells exposed to IKK16 or SGI1027. Upon the staining with CalceinAM and Endoplasmic Reticulum (ER) tracker, the results showed low green fluorescence, suggesting compromised cellular membrane integrity and dilated ER, respectively, compared to untreated cells (Fig. 2h).

In another experiment, we treated KAIMRC2 cells with known apoptosis-inducing drugs, Mitoxantrone, Staurosporine, Daunorubicin, Doxorubicin, and Etoposide alongside IKK16, SGI1027 and Taxol. The cells were stained afterward with YoProI, a fluorescent marker for apoptotic cells. Our results show that the cells treated with IKK16, SGI1027 and Taxol showed minimal staining for YoProI, as compared to other drugs (Fig. 4a).

### Gene expression profiling of cells induced by IKK16 and SGI1027

To profile gene expression of paraptosis versus apoptosis, KAIMRC2 cells were treated with IKK16, SGI1027, or Mitoxantrone. The effect of the compounds on gene expression was assessed using RT-qPCR. We found several upregulated and downregulated genes with IKK16 and SGI1027 compared to mitoxantrone. In particular, the BCL-2 family gene, BCL2L10, tumor necrosis factor superfamily member 10 (TNFSF10), and Tumor protein 73 (TP73) genes were significantly upregulated in KAIMRC2 cells treated with IKK16 or SGI1027, whereas Growth Arrest and DNA Damage (GADD45A) was significantly downregulated (Fig. 5).

Regarding cellular function, downregulated genes clustered into those involved mainly in the cellular response to chemokine, apoptotic signaling pathway, and cellular stress. Additional RT^2^ profiler PCR arrays were used to identify differential expression genes in KAIMRC2 cells treated with IKK16. A total of 30, 35, and 21 genes were identified from the human stress and toxicity array (Fig. 5c), osmotic stress array (Fig. 5d), and cell death array (Fig. 5b), respectively. Among them, Endothelin 1 (EDN1), GADD45A, were substantially downregulated whereas interferon gamma (IFNG), and tumor necrosis factor (TNF) expressions wereunexpectedly downregulated when the cells were treated with IKK16. The threshold was set as log2 fold change (FC) compared with untreated cells. To get a better understanding of the molecular mechanisms of paraptosis, a functional network analysis for the differentially expressed genes was constructed. Supplementary Fig. S2 shows two networks. One is for downregulated genes, and the other highlights the upregulated genes in KAIMRC2 cells undergoing paraptosis.

The down-regulated genes are involved in chemokines response, apoptotic signaling pathway regulation, and TNF response and the upregulated genes are mostly involved in cellular response to environmental and external stimuli. The FDR is less than 5%.

### Proteomics analysis of IKK16 treated KAIMRC2 cells

The initial stage of our study employed proteomics analysis to elucidate the potential role of IKK16 in inducing paraptosis within the KAIMRC2 breast cancer cell line. This was achieved by performing label-free protein quantification using Triple TOF MS/MS operated in DDA. In addition to IKK16, KAIMRC2 cells were exposed to Mitoxantrone and SGI1027, known to induce apoptosis, and to DMSO as negative controls. Throughout all experimental samples, a total of 1270 proteins were identified. Notably, 35% of these proteins were shared across all samples, as shown in Fig. 5a. The IKK16 sample exhibited more uniquely identified proteins (*n* = 104) than the other samples analyzed. Global quantitative proteomics analysis demonstrated a high degree of reproducibility for the three technical replicates with an average coefficient of variation (CV) of less than 14%. Furthermore, quantitative analysis of the two experimental replicates across all drug samples showed a high correlation, reflected by R-squared values exceeding 0.90 (Fig. 6b). Supplementary Fig. S1 illustrates the reproducibility assessment of protein quantification for the remaining control and drug samples.

Differentially expressed protein analysis was conducted using a pairwise comparison approach, comparing the DMSO control group to each drug sample group (IKK16, Mitoxantrone, and SGI1027). Differentially expressed proteins were defined using pre-defined statistical criteria. A fold change threshold of ≥1.5 for upregulation and ≤0.66 for downregulation was applied, with a *p*-value significance level of *p* < 0.05. The quantitative results were visualized using a volcano plot, as exemplified by the pairwise comparison of DMSO and IKK16 samples (Fig. 6c). Volcano plots for the remaining comparisons of proteins are in supplementary Fig. S1. A comparison of DMSO and IKK16 identified 46 significantly differentially expressed proteins, 28 upregulated and 18 downregulated proteins. 236 significantly differentially expressed proteins were identified between DMSO and Mitoxantrone. In contrast, a comparison between DMSO and SGI1027 identified a single protein, Thymidylate synthase (TYMS), with a statistically significant upregulation. Following the identification of differentially expressed proteins, the ShinyGO tool was employed to elucidate their potential enrichment in specific biological pathways using the Reactome database. Pathways enrichment analysis revealed significant enrichment of the top three pathways in DMSO/IKK16 samples, notably Unwinding of DNA, Cholesterol biosynthesis, and DNA strand elongation (Fig. 6d). In DMSO/ Mitoxantrone samples, Pathways enrichment analysis identified significant enrichment for peptide chain elongation, viral mRNA Translation, and Eukaryotic Translation Termination.

To validate the differential expression of key proteins identified by our proteomics analysis, we performed western blotting for eleven proteins exhibiting a statistically significant change in abundance (Fig. 6f). Following were the proteins: Glucose transporter 1 (GLUT1), facilitates the transport of glucose across the plasma membrane [45], lanosterol synthase (LSS), a key enzyme in the cholesterol biosynthesis pathway [46], Farnesyl-Diphosphate Farnesyltransferase 1 (FDFT1), metabolic pathway regulator in cancer [47] and a proposed ferroptosis marker [48], Annexin α6, regulates the release of Ca^2+^ and receptor localization [49], Stearoyl-CoA desaturase (SCD), plays an important role in lipid biosynthesis [50], Aldehyde dehydrogenase 1 family member B1 (ALDH1B1), involved in alcohol metabolism and promotes cancer [51], Growth/differentiation factor 15 (GDF15), a hormone produced in response to stress and regulates growth [52], Isopentenyl-diphosphate Delta-isomerase 1 (IDI1), a cytoplasmic enzyme involved in the biosynthesis of isoprenoids including cholesterols [53], Sequestosome 1 (SQSTM1), an autophagosome cargo protein, plays a pivotal role in stress and aging [54], Annexin α2, a regulator of endogenous low-density lipoprotein receptor levels [55], and, Prothymosin alpha (PTMA), an antiapoptotic protein, binds to p8 to regulate apoptosis [56]. Figure 6g is the bar graphical representation of the WB in Fig. 6f. A string network using Cytoscape was also drawn to find the connections between proteins known as the targets of IKK16 and SGI 1027 and the identified proteins using proteomics and WBs. The string network revealed that most of the identified proteins were linked with each other through cJUN, SQSTM, PARP and PTEN.

### Immunofluorescence-based verification of the selected protein candidates in different cancer cell lines

To verify the results of the proteomics analysis, we conducted a fluorescence-based assay to assess the expression levels of proteins in three cell lines, KAIMRC2 (Fig. 7a), MDAMB231 (Fig. S3a), and HCT8 (Fig. S3b). The cells were exposed to IKK16 and compared to control cells. Most identified proteins were upregulated upon exposure to IKK16, even though the expression levels of proteins in the cell lines differed. Interestingly, ALIX is upregulated in our results, which suggests that ALIX is overexpressed to counter the paraptosis induction and help cells escape cell death. The low dosage of ROS as a signaling molecule can activate poly (ADP-ribose) polymerase (PARP) [57] and has a role in paraptosis in the absence of caspase activity. Our results indicate an increase in PARP protein using immunofluorescence. It has been shown that the reduction of Olig2 increased apoptosis in melanoma cells [58]. Our results showed increased Olig2 expression after treatment with IKK16, suggesting that IKK16 does not trigger apoptosis.

### Overexpression of GADD45A and NPC2 mitigates IKK16- and SGI1027-induced paraptosis in cancer cells

**Fig. 8:**
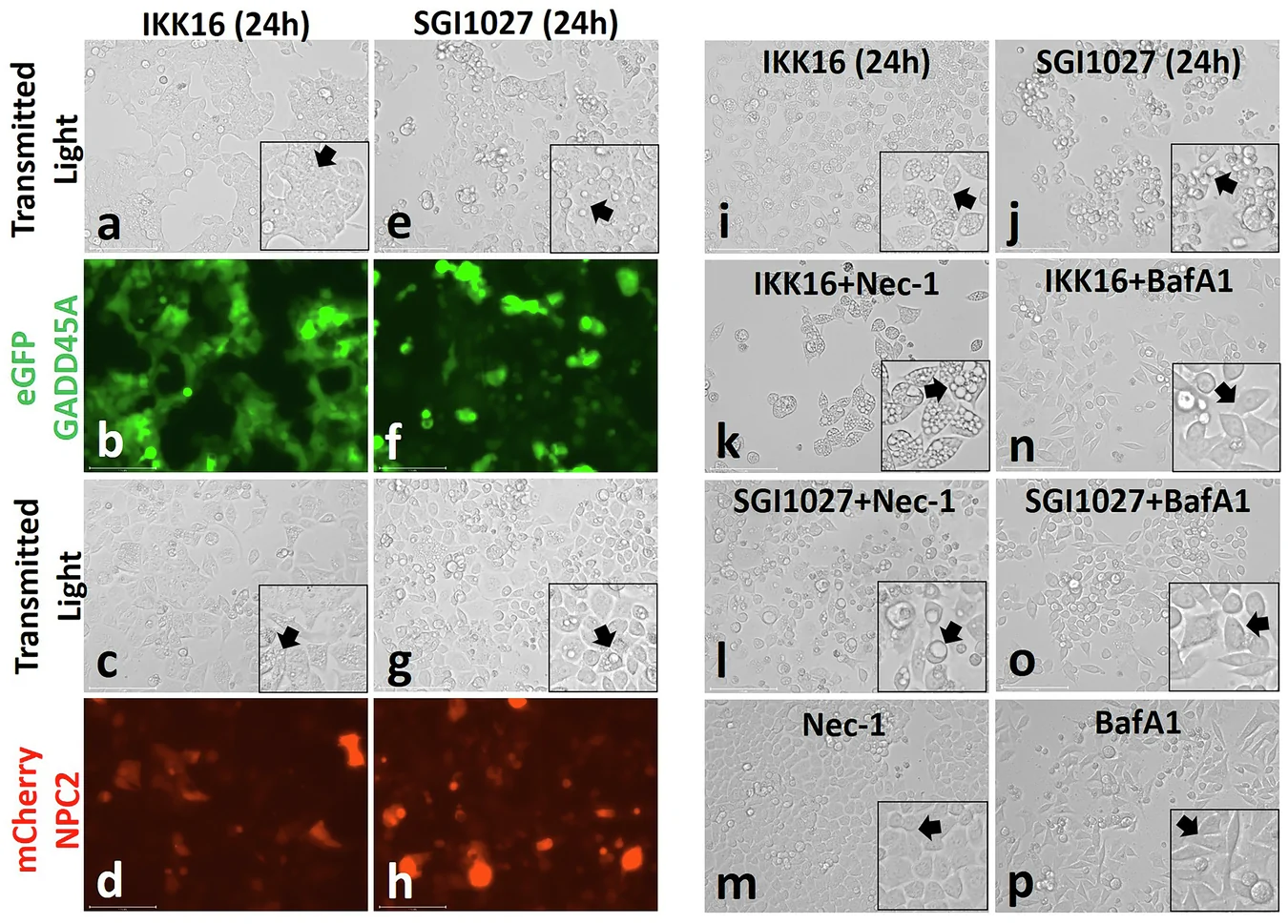
20× images of stable KAIMRC2 cell line transfected with. **a**, **b**, **e**, **f** GADD45A-eGFP and **c**, **d**, **g**, **h** NPC2-mCherry. Cells were treated with (**a**–**d**, **i**) 6.25 µM IKK16, and (**e**–**h**, **j**) 12.5 µM SGI1027 for 24 h. The overexpression of GADD45A and NPC2 resulted in reduced vacuole formation and increased cell proliferation, indicating a crucial role for these proteins in cellular vacuolation. The decrease in vacuole formation was more prominent in GADD45A-transfected cells than in NP2-transfected cells. This result aligns well with our gene expression and Proteomics data, which showed downregulation of GADD45A and NPC2 after treatment with IKK16. **k**–**n** Treatment with Autophagy Blockers and Necroptosis Inhibitors. KAIMRC2 cells were treated simultaneously with (**k**, **n**) 6.25 µM IKK16 and Nec-1 (**l**, **o**) 12.5 µM SGI1027 and 6.25 µM Nec-1 (**m**) 6.25 µM Nec-1 only and (**p**) 0.1 µM BafA1 for 24 h. Post-24 h, transmitted-light phase-contrast images were acquired using an EVOS M7000 microscope. BafA1 was able to inhibit partially vacuole formation in both SGI1027 and IKK16 -treated cells. Whereas Necrostatin1 was not able to inhibit vacuole formation in SGI1027 and IKK16 -treated cells. Similar results were observed when the cells were treated with vacuole-inducing compounds SGI1027 and IKK16 for 24 h, followed by the treatment with Bafilomycin A1 and Necrostatin-1. Scale bar = 125 µm.

We hypothesized that the upregulation of individual or combination of genes downregulated in paraptotic KAIMRC2 cells, post-IKK16 treatment could mitigate IKK16 paraptotic activity. From the gene expression profiles and proteomic experiments, GADD45A and NPC2 appeared to be the most markedly downregulated genes; and hence we generated KAIMRC2 cells stably expressing GADD45A-eGFP or NPC2-mCherry. Similarly to the earlier experiments, parent KAIMRC2 cells, GADD45A-eGFP-, and NPC2-mCherry-expressing cells were treated with 6.25 μM IKK16 or 12.5 μM SGI1027 and monitored the cells for 24 h (Fig. 8). Cytoplasmic paraptotic vacuoles were visible in the wild-type KAIMRC2 cells at 24 h when treated with IKK16, or SGI1027. However, when compared to the wild-type cells, the vacuoules were noticeably near absent with IKK16 and SGI1027 treatments in the GADD45-GFP-expressing cells, and smaller in the NPC2-mCherry-expressing cells (Fig. 8a–h). both overexpressing lines showed better viability than the parent KAIMRC2 line under the IKK16 and SGI1027 conditions.

### A combination of IKK16 and SGI1027 with chemotherapeutic drugs enhances cell death in cancer cells

Strikingly, combining these small-molecule inhibitors with the anthracyclines resulted in a profound, dose-dependent decrease in cell proliferation that was significantly more potent than any single-agent treatment. To assess the effectiveness of a combinational approach to target cancer cells, KAIMRC2 cells were treated with IKK16 and SGI1027 alone or in combination with chemotherapeutic drugs such as Mitoxantrone, Daunorubicin, Doxorubicin, paclitaxel (Taxol), and Staurosporine.

Briefly, to perform combination treatment in a 96-well plate format, cells were treated with 3.12 µM IKK16 or 6.25 µM SGI1027 only to induce cytoplasmic vacoulization for 24 h. Then, chemotherapeutic drugs Mitoxantrone, Daunorubicin, Doxorubicin, Taxol, and Staurosporine were added to the vacoulated cells at graded concentrations (3.12, 1.56, 0.78, and 0.4 µM) for another 48 h followed by the MTT cell viability assay.

**Fig. 9:**
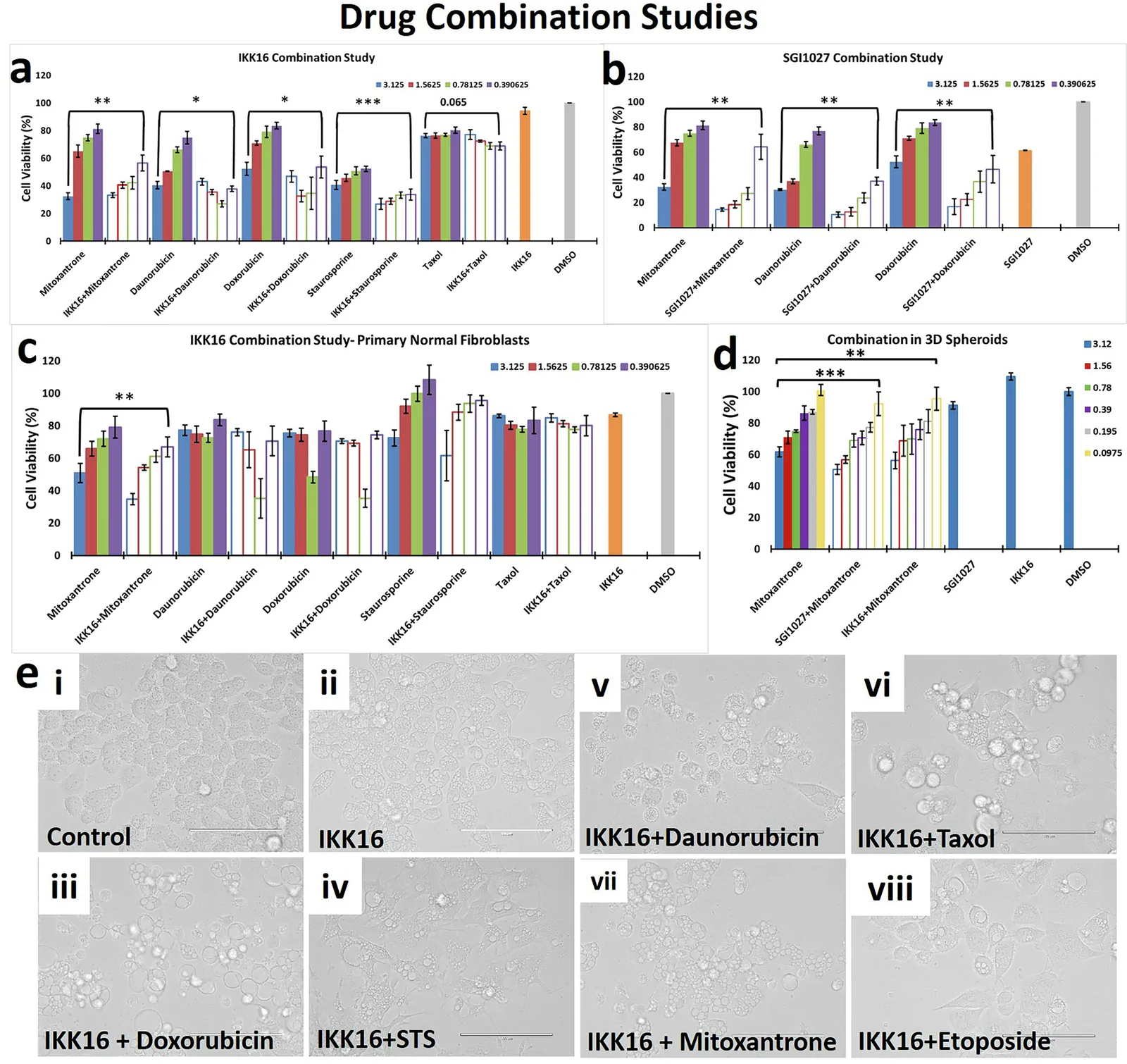
Combinational therapy of IKK16 and SGI1027 with other drugs increased their potency. **a** Cell viability of KAIMRC2 cells treated with 3.12 µM IKK16 or (**b**) 6.25 µM SGI1027 in combination with other chemotherapy drugs at graded concentrations for 72 h, followed by the cell viability testing. Cell viability was normalized to the untreated cells. **c** Cell viability of Primary Normal Fibroblasts treated with IKK16 in combination with other chemotherapy drugs. Cell viability was normalized to the untreated cells. **d** 3D KAIMRC1 spheroids were treated with IKK16 and SGI1027 in combination with chemotherapeutic drug Mitoxantrone to evaluate the effect of these compounds in a more complex 3D cell culture. Our results show a similar trend to that observed in 2D cell cultures. **e** i–viii Representative microscopic images of KAIMRC2 cells treated with IKK16 for 24 h. It is noted that the addition of low dose of IKK16 can increase cancer cell death compared to cells treated with chemotherapeutic drugs alone. *P*-values: * = 0.05, ** = 0.01, *** = 0.001.

Our results showed that cell viability noticeably decreased when IKK16 or SGI1027 was combined with other chemotherapeutic drugs. This indicates a synergistic effect on cell death compared to each treatment alone (Fig. 9a, b). Our results show that even deficient concentrations of chemotherapeutic drugs can be combined with IKK16 and SGI1027 to induce significant killing in the cancer cells. Figure 9e depicts the effect of different treatments on the cells. Interestingly, normal primary fibroblasts did not show any considerable killing when the drugs were combined except for Mitoxantrone combination, which might be due to the potency of Mitoxantrone (Fig. 9c). Moreover 3D KAIMRC1 spheroids were treated with IKK16 and SGI1027 in combination with chemotherapeutic drug Mitoxantrone to evaluate the effect of these compounds in a physiologically relevant 3D cell culture system. Our results show a similar trend to that observed in 2D cell cultures.

We validated our results using an open-source web-based application, Synergy Finder (07.09.2024-R-3.10.3). This application is used for analyzing drug combination dose-response matrix data [59]. The Supplementary Fig. S4 shows dose response heat maps, synergy maps [60] and sensity maps [61] of two drug combinations for both IKK16 and SGI1027 combined with chemotherapeutic drugs. Highest single agent (HAS) model was used to analyze drug combination. The HSA model considers the expected combined effect to be the maximum of a single drug response at the corresponding concentration. IKK16 and SGI1027 were used at a fixed concentration to induce cytoplasmic vacuolization, and the chemotherapeutic drugs were used at graded concentrations. The calculated synergy scores under −10 indicate antagonism, from −10 to 10 indicate additivity, and above 10 indicate synergism. Only the combination of Taxol with IKK16 resulted in a synergy score below 10 indicating additivity which is because both the drugs induce paraptosis in the cells.

## Discussion

### Overcoming apoptotic resistance via paraptotic proteostasis disruption

The development of intrinsic or acquired resistance to apoptosis remains a primary barrier to successful cancer chemotherapy [62]. Because traditional cytotoxic agents rely heavily on intact caspase cascades and functional *p53* signaling to trigger apoptosis, mutations within these pathways frequently render cancer cells chemoresistant. To circumvent this hurdle, exploring alternative, non-apoptotic cell death modalities has emerged as a promising therapeutic frontier [63–69]. In this study, we investigated a novel sequential combination strategy utilizing two small molecules with distinct primary targets: IKK16, an IκB kinase inhibitor disrupting inflammatory signaling [70], and SGI1027, a DNMT inhibitor targeting epigenetic regulation [71].

Despite their disparate molecular initiation points, our phenotypic and proteomic data demonstrate that both compounds funnel into a shared cellular stress response. Both agents disrupt cellular proteostasis, overwhelm the endoplasmic reticulum (ER), and perturb calcium homeostasis, culminating in extensive cytoplasmic vacuolization and ATP depletion. Crucially, because this paraptotic cascade executes independently of caspase activation, it successfully bypasses the genetic blocks that typically shield cancer cells from apoptosis-inducing therapies.

### Transcriptomic remodeling and the paraptotic signature

Our gene expression profiling revealed a distinct transcriptomic signature that explicitly distinguishes paraptosis from classical apoptosis. A pivotal finding was the significant downregulation of *GADD45A* in paraptotic cells. Under standard genotoxic stress, *GADD45A* is typically upregulated, activating *p38* and *p53* to drive apoptosis [72]. However, in our model, the repression of *GADD45A* appears to act as a molecular switch, diverting cells away from apoptotic induction and placing them into a state of cellular senescence characterized by altered morphology and compromised structural integrity. Overexpression of *GADD45A* or *NPC2* partially mitigated vacuolization and restored cell proliferative capacity. The incomplete nature of this rescue indicates that paraptosis is governed by a complex, multifactorial regulatory network rather than a single dominant effector. Nevertheless, these functional validation experiments confirm that *GADD45A* and *NPC2* are central biological modulators of the paraptotic phenotype rather than passive bystander markers, positioning *GADD45A* as a robust candidate biomarker for paraptosis.

This paraptotic state is further corroborated by the collective downregulation of *IFNG* and its downstream target *IL8*, confirming that classical apoptotic machinery is not the primary driver of cell death in IKK16-treated cells. Interestingly, while the apoptosis-inducing agent Mitoxantrone expectedly repressed the anti-apoptotic gene *BIRC5* (Survivin) to sensitize KAIMRC2 cells to DNA damage [73], *BIRC5* was marginally upregulated following IKK16 and SGI1027 exposure. This variation reinforces the independence of the paraptotic pathway from standard survival-factor suppression. Concurrently, the significant upregulation of *TNFSF10* (TRAIL) aligns with recent evidence demonstrating that TNF receptor superfamily members, such as TNFRSF19, actively promote ER stress-induced paraptosis [74]. Furthermore, the marked downregulation of *EDN1*, a gene whose suppression is traditionally tied to apoptosis [75], suggests that while apoptosis and paraptosis operate through independent execution mechanisms, they engage in cross-talk at shared upstream regulatory junctions.

### Exploiting metabolic vulnerabilities and dual-edged synergies

At the proteomic level, we observed a pronounced overexpression of the glucose transporter *GLUT1* (SLC2A1) in paraptotic cells. While cancer cells typically upregulate *GLUT1* to fuel glycolysis, this alterations presents a unique therapeutic vulnerability. Recent literature indicates that elevated levels of related transporters (such as *SLC7A11*) under glucose-starved conditions cause an abnormal accumulation of intracellular disulfide molecules, inducing aberrant cross-linking among actin cytoskeletal proteins and catastrophic network collapse [76]. Our findings suggest that exposing glucose-deprived cancer cells to low-dose paraptosis inducers like IKK16 or SGI1027 could severely compromise structural integrity, offering a potent metabolic strategy to arrest proliferation and drive targeted cell death.This metabolic and structural destabilization underpins the success of our proposed “dual-edged sword” combination strategy. By using low, sub-toxic doses of paraptosis-inducing drugs, we successfully induce a state of reversible cytoplasmic vacuolization and mitochondrial membrane depolarization, depleting cellular ATP. This bioenergetic crisis critically sensitizes the tumor cells. When followed sequentially by a low dose of standard chemotherapy, these weakened cells are pushed over the survival threshold into apoptosis or necrosis.

### Clinical relevance and future directions

From a clinical translation perspective, determining the correct dosage, ratio, and scheduling remains the most challenging aspect of combination therapy design. However, the therapeutic window achieved by our low-dose sequential protocol offers a compelling advantage: by maximizing antitumor efficacy at minimized concentrations, this approach may allow surrounding healthy tissues to recover more efficiently from chemotherapeutic shock, potentially reducing systemic toxicities.

While our study provides robust proof-of-concept validation of this paraptotic-apoptotic synergy across both 2D and 3D in vitro culture architectures, translating these findings to the clinic requires further steps. Future work must focus on evaluating this combination regimen in vivo using relevant animal models to assess pharmacokinetic profiles, confirm tumor microenvironment penetration, and establish comprehensive systemic toxicity profiles.

## Conclusion

In conclusion, we show that IKK16 and SGI1027 can induce cytoplasmic vacuolization, leading to cell paraptosis. Interestingly, at low concentrations of these compounds, cytoplasmic vacuolization can also be reversed and does not necessarily kill the cells. We have also identified new gene and protein markers of paraptosis. Our data explored the therapeutic advantage of combining these two mechanistically distinct pathways by combining paraptosis-inducing compounds with chemotherapeutic drugs. Both compounds bypass classical apoptosis by inducing paraptosis in cancer cells, making them highly effective against apoptosis-resistant malignancies. These findings open up new possibilities for developing targeted combination therapies that exploit alternative cell death pathways, providing a promising approach to counter chemoresistance in prevalent global malignancies.

## Supplementary information

Supplementary Material File

Supplementary Figure S1

Supplementary Figure S2

Supplementary Figure S3

Supplementary Figure S4

Original Data

## Data Availability

All the relevant data are available in the manuscript. Any further data are available from the authors upon reasonable request and with permission of King Abdullah International Medical Research Center.
