# Supplementary material for: IKK16 and SGI1027 induce cytoplasmic vacuolization, and their combination with chemotherapy drugs can enhance the efficacy of cancer therapeutics
Source: Cell Death Dis. 2026 Aug 5;17(1):954. doi: 10.1038/s41419-026-09142-w (PMC13748669; doi:10.1038/s41419-026-09142-w)
Supplement: Supplementary file 1 [file 41419_2026_9142_MOESM1_ESM.docx]

**Supplementary Material File**

**Supplementary Figure S1: The scatter plots visualize the reproducibility of protein intensity measurements across two replicate experiments.** (a) Mitoxantrone, (b) SGI1027, (c) DMSO. Volcano plots for differentially expressed proteins (d) Mitoxantrone/DMSO, (e) SGI1027/DMSO. Red dots represent upregulated proteins (fold change ≥1.5), blue downregulated proteins (fold change ≤ 0.66), and gray dots are not significant and not regulated proteins. p-value significance level was set as 0.05 (f) Hierarchical clustering of differentially expressed proteins in KAIMRC2. Cells treated with SGI 1027, IKK 16, or Mitoxantrone versus DMSO as a control. Red represents upregulated proteins, while blue represents downregulated proteins.

**Supplementary Figure S2:** The scatter plots visualize the reproducibility of gene intensity measurements across two replicate experiments. Volcano plots for differentially expressed genes (a) Cell Death, (b) Stress and Toxicity Array, (c) Osmotic Stress Array. Dots represent up- or down-regulated proteins (fold change ≥1.5), and (d) Pathway analysis of significantly up- or down-regulated genes.

**Supplementary Figure S3: Validation of proteomic data by Immunocytochemistry.** (a) cLSM images of MDAMB231 and HCT8 cells stained for selected protein candidates. The protein of interest is shown in green and the nucleus is in blue. The images show staining of the following proteins: CD133, Glut1, LSS, FDFT1, Annexin α6, SCD, ALDH1B1, GDF15, IDI1, SQSTM1, Annexin α2, PTMA, ALIX, Olig2, PARP, LC3A/B  (b) Graphical representation of relative expression for candidate proteins using averaged fluorescence intensities. P-values: *=0.05, **=0.01, ***=0.005

**Supplementary Figure S4:**  Synergistic Effect demonstration using the Synergy Finder Web application. It shows dose response heat maps, synergy maps and sensitivity maps of two drug combinations for both IKK16 and SGI1027 with chemotherapeutic drugs.
