# Supplementary material for: IKK16 and SGI1027 induce cytoplasmic vacuolization, and their combination with chemotherapy drugs can enhance the efficacy of cancer therapeutics
Source: Cell Death Dis. 2026 Aug 5;17(1):954. doi: 10.1038/s41419-026-09142-w (PMC13748669; doi:10.1038/s41419-026-09142-w)
Supplement: Supplementary file 2 [file 41419_2026_9142_MOESM6_ESM.docx]

**ANTI CARD9-ANNOTATED**

**ANTI CAV1-ANNOTATED**

**ANTI CCND1 Annotated**

**ANTI ERBB2-ANNOTATED**

**ANTI JUN Annotated**

**ANTI LC3 Annotated**

**Anti p-p38-Annotated**

**ANTI pPTEN-Annotated**

**ANTI-ACTIN CARD9 PTEN Annotated**

**ANTI-ACTIN LC3 Annotated**

**ANTI-ACTIN-ERBB2 JUN ANNOTATED**

**ANTI-ACTIN-p-p38-Annotated**

**ANTI ALDH1B1-ANNOTATED**

**ANTI GDF15-ANNOTATED**

**ANTI-bACTIN Annotated**

**ANTI-bACTIN Annotated**

**ANTI-bACTIN Annotated**

**ANTI-ANNEXIN ANNOTATED**

**ANTI-CD133-ANNOTATED**

**ANTI-FDFT-1 ANNTATED**

**ANTI-LSS Annotated**

**ANTI-SQSTM1 ANNOTATED**
